# Effect of Water Compressibility, Sea-floor Elasticity, and Field Gravitational Potential on Tsunami Phase Speed

**DOI:** 10.1038/s41598-019-52475-0

**Published:** 2019-11-14

**Authors:** Ali Abdolali, Usama Kadri, James T. Kirby

**Affiliations:** 1 National Oceanic and Atmospheric Administration (NOAA), College Park, MD 20740 USA; 2 University Corporation for Atmospheric Research (UCAR), Boulder, CO 80301 USA; 3 School of Mathematics, Cardiff University, Cardiff, CF24 4AG UK; 4 Department of Mathematics, Massachusetts Institute of Technology, Cambridge, MA 02139 USA; 5 Department of Civil and Environmental Engineering, University of Delaware, Newark, DE 19716 USA

**Keywords:** Natural hazards, Physical oceanography, Geodynamics, Geophysics, Applied mathematics

## Abstract

Tsunamis can propagate thousands of kilometres across the ocean. Precise calculations of arrival times are essential for reliable early warning systems, determination of source and earth properties using the inverse problem, and time series modulation due to frequency dependency of phase speed. Far field observatories show a systematic discrepancy between observed and calculated arrival times. Models in present use and based on incompressible hydrodynamics and interaction with a rigid ocean floor overestimate the phase speed of tsunamis, leading to arrival time differences exceeding tens of minutes. These models neglect the simultaneous effects of the slight compressibility of water, sea-bed elasticity, and static compression of the ocean under gravity, hereinafter gravity. Here, we show that taking these effects into account results in more accurate phase speeds and travel times that agree with observations. Moreover, the semi-analytical model that we propose can be employed near real-time, which is essential for early warning inverse models and mitigation systems that rely on accurate phase speed calculations.

## Introduction

The vast majority of conventional ocean circulation models treat the sea as an incompressible medium (ignoring the compression of the at-rest water column due to its own weight as well as acoustic contributions to motion) overlying a rigid sea-floor^1–3^. These assumptions are valid when the time periods are relatively short, and the corresponding phase speeds are low. It has been argued in various studies that the contributions of water compressibility and earth elasticity are vital not only for proper description of surface gravity waves^4–8^, but even more for waves travelling in the earth crust, such as P, S, Rayleigh and Love waves^9^, and acoustic-gravity waves that propagate in the water column^10–16^, or couple with the sea-floor^17^. The latter travel at speeds that far exceed the maximum tsunami phase speed carrying information on the fault geometry and dynamics^15^, and thus could be employed for early tsunami warning systems^18^ and inverse models^19^, if analysed real-time.

**Figure 1:**
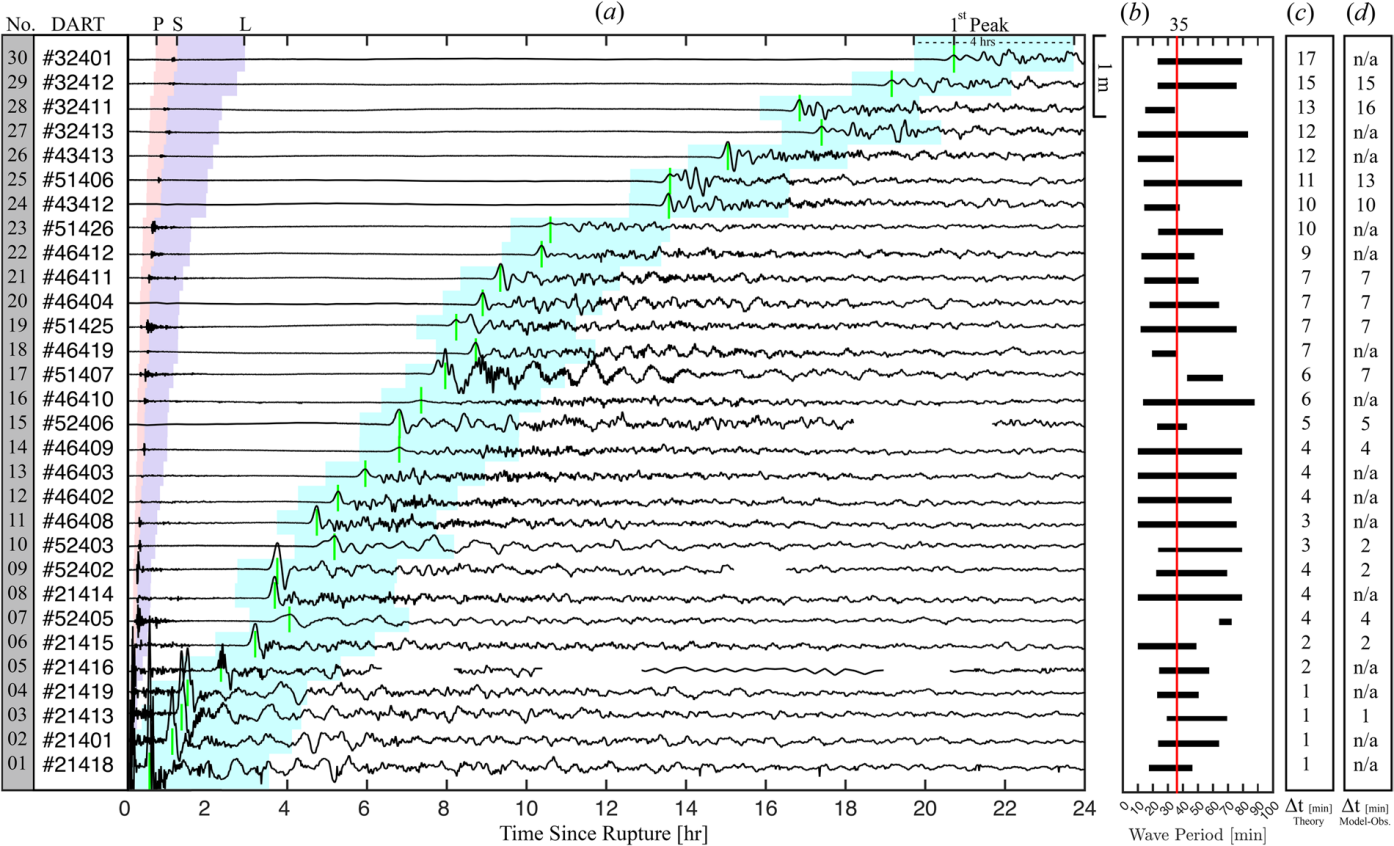
(**a**) The time series at DART observatories (From top to bottom sorted by the furthest to nearest to the epicenter, shown by the yellow star - Fig. 3). The approximate arrival time of compression waves in solid (P), shear waves in solid (S) and compression waves in water (L) and first peak arrival (green) are shown; (**b**) The range of wave period in a 4 hrs time window near the 1st peak (1 hr before, 3 hrs after). Red line (35 min) represents the mean for all stations, which is used for a sample computation, shown in Fig. 3; (**c**) that shows the discrepancy between the computed arrival time of tsunami waves from the full solution (Eq. 1) and standard dispersion relation for the case of incompressible ocean over rigid bottom as shown in Fig. 3 and (**d**) earlier arrival of the signals extracted from the incompressible models^26,27,33,34^ relative to the observations.

Tsunami waves have been extensively studied in recent years, with attention focused on various physical features including thermal or salinity-based density stratification^6,20^, compressibility of the water column^4,6,8,15,20–24^, elastic deformation of the underlying solid earth^17,20,25^ or combined effect of compressibility, stratification, and elasticity^26^. These studies confirmed, independently, that the time lag between model output and observations is sensitive to many of the physical features virtually neglected. Consequently, the time series at far field observatories are shifted in order to match arrival of waveforms extracted from these models and observations (see Figs 1d and 2 of ref.^5^ and Fig. 3 of ref.^27^). In addition, it has been observed that the arrival delay is taking place in deep ocean where nonlinear effects are negligible^25^ and the aforementioned parameters are dominant.

**Figure 2:**
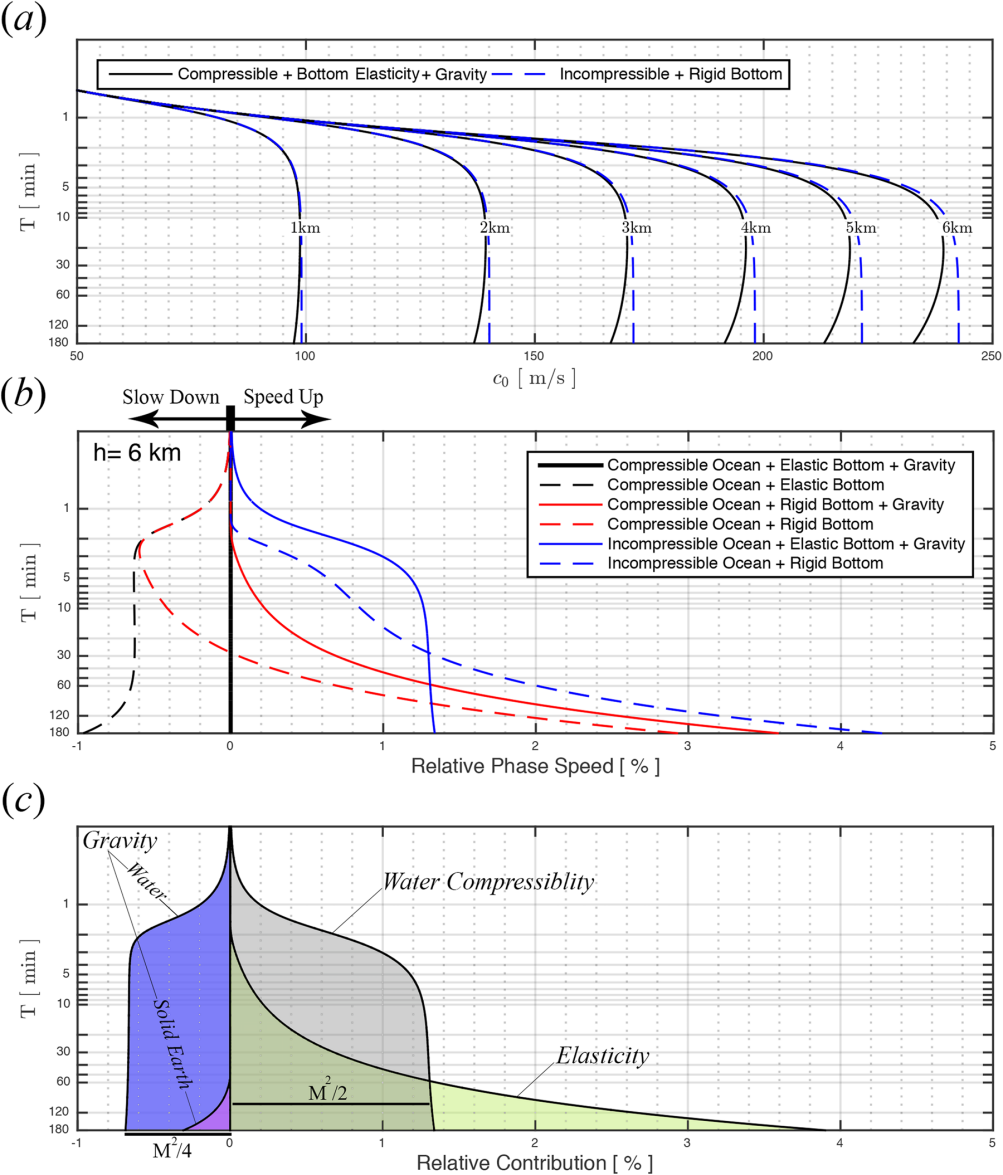
(**a**) Phase speed for varying water depth (1 km increments) for a compressible ocean overlying an elastic bottom with background density in water and solid earth (solid lines) and an incompressible ocean overlying a rigid bottom (dashed blue line); (**b**) The percentage of phase speed increase/decrease relative to the case of a compressible ocean overlying an elastic bottom with background density in water and earth (solid black) for 6 km water column. The relative phase speed for the case of compressible ocean with an elastic bottom (dashed black), compressible ocean overlying a rigid bottom with background density (solid red), compressible ocean overlying a rigid bottom (dashed red), an incompressible ocean over an elastic bottom with background density (solid blue), an incompressible ocean over a rigid bottom (dashed blue) and an incompressible ocean over a rigid bottom with background density (solid red) are shown; (**c**) The relative contribution of water compressibility, bottom elasticity and background density on phase speed increase/decrease for 6 km water column are shown by shaded areas. M = $\sqrt{gh}/{c}_{l}$ is a Mach number based on the linear incompressible surface long wave speed.

In this study, we use potential theory to evaluate the effects of compressibility, elasticity and gravity on the calculated phase speed of tsunami. More specifically, water is treated as an inviscid, barotropic fluid with constant sound speed, ${c}_{l}$, and fluid motion is assumed to be irrotational. On the other hand, the solid layer is treated as an elastic half space that undergoes rotation and compression with constant pressure and shear wave speeds, ${c}_{p}$ and ${c}_{s}$. The problem at hand can be expressed by three wave equations governing a velocity potential ${\phi }_{l}$ in water, and a dilatation potential ${\phi }_{s}$ and rotation potential ${\psi }_{s}$ in the solid sea-floor, given in dimensionless form by

1 $$\begin{array}{l}{\nabla }^{2}{\phi }_{l}-2{\gamma }_{l}({\phi }_{l,tt}+{\phi }_{l,z})=0,\qquad -\,1\le z\le 0;\\ {\nabla }^{2}{\phi }_{s}-2{\gamma }_{s}({\phi }_{s,tt}+{\phi }_{s,z})=0,\qquad z\le -\,1;\\ {\nabla }^{2}{\psi }_{s}-2{\gamma }_{s}({\psi }_{s,tt}+{\psi }_{s,z})=0,\qquad z\le -\,1,\end{array}$$

where ${\gamma }_{i}=gh/2{c}_{i}^{2}$, (with $i=l,p,s$) are small dimensionless parameters representing the squares of the ratios of surface gravity waves to sound, pressure and shear wave speeds. respectively. All quantities in Eq. (1) are normalized using the water depth $h$ as a length scale, $\sqrt{h/g}$ as a timescale, and densities are normalized by the water density ${\rho }_{l}$, (see supplementary file for dimensional analysis). The solution for linearized motion of plane progressive waves in a horizontally uniform domain then follows from applying kinematic and dynamic boundary conditions at the free surface, matching normal stress and displacements at the ocean-seabed interface, and requiring the dilatation and rotation potentials vanish at $z\to -\,\infty$ (see supplementary material for details). The full dispersion relation for the prescribed frequency $\omega$ is then given by

2 $$r\tanh (r)=\frac{{\omega }^{2}\left({\varepsilon }_{1}+{\varepsilon }_{2}\right)}{{\varepsilon }_{1}+{\varepsilon }_{2}{\omega }^{4}/{r}^{2}+\beta {\gamma }_{l}/{r}^{2}},$$

where ${r}^{2}={k}^{2}-2{\gamma }_{l}{\omega }^{2}+{{\gamma }_{l}}^{2}$ is the eigenvalue for the vertical structure of ${\phi }_{l}$ in the water, $k$ is the wavenumber in the horizontal direction, and ${\varepsilon }_{1}$, ${\varepsilon }_{2}$ and $\beta$ represent the elasticity and gravity effects defined in Eqs (3)–(5). The phase speed is then computed by ${c}_{0}=\omega /k$, where $k$ dictates the eigenvalues $r$ (see Eq. 6). Note that ${k}^{2} > 0$ and ${r}^{2} > 0$ corresponds to the progressive gravity mode ($n=0$) which decays exponentially with depth. Propagating non-evanescent acoustic-gravity waves can rise under the special condition where $r$ is imaginary, yet $k$ is real. Unlike the discrete spectrum of the progressive gravity waves, the spectrum of trapped modes, exponentially decaying in $(x,y)$, is continuous where ${k}^{2} < 0$. However, there is no interaction between progressive and trapped modes in a spatially uniform domain. The general dispersion relation (2) accounts for the effects of compressibility, elasticity, and gravity. It turns out that ignoring any combination of these effects manipulates the phase speed of the propagating surface wave (Fig. 2 and Table 1). Specifically, ignoring elasticity or compressibility results in an overestimate of the phase speed, whereas ignoring gravity, both within water and sea-floor, results in an underestimate. Note that neglecting ${\gamma }_{i}$ the dispersion relation Eq. (17) of ref.^17^ is retrieved, whereas if elasticity is ignored, retaining water compressibility and gravity terms, the dispersion relation Eq. (3.2) of ref.^28^ or Eq. (11) of ref.^4^ are retrieved (${\varepsilon }_{1}=1;{\varepsilon }_{2}=0$). Neglecting elasticity and gravity leads to the standard dispersion relation ${\omega }^{2}=r\tanh (r)$. The phase speeds corresponding to all possible combinations of compressibility, elasticity and gravity are depicted in Table 1 and Fig. 2. It is worth noting that the surface gravity wave (mode $n=0$) has a cut-off frequency of $0\ Hz$, which allows propagation of the plane wave at any excitation frequency. Thus, for tsunamis within the frequency range of a few minutes to a few hours, waves are progressive with dominance of the discrete spectrum. Alternatively, a continuous spectrum should be considered when ${k}^{2} < 0$, where the modes become evanescent decaying exponentially in depth and horizontal plane. This case is relevant only in the near field, within a range of several water depths^29,30^.

**Table 1:** Dispersion relations for different combinations of compressibility, elasticity and gravity effects in the water column.

#	Compressibility	Elasticity	Gravity	Dispersion Relation	${k}^{2}$
(1)	✓	✓	✓	$r\,\tanh (r)=\frac{{\omega }^{2}\left({\varepsilon }_{1}+{\varepsilon }_{2}\right)}{{\varepsilon }_{1}+{\varepsilon }_{2}{\omega }^{4}/{r}^{2}+\beta {\gamma }_{l}/{r}^{2}}$	${r}^{2}+2{\gamma }_{l}{\omega }^{2}-{\gamma }_{l}^{2}$
(2)	✓	✓	⨯	$r\,\tanh (r)=\frac{{\omega }^{2}\left({\varepsilon }_{1}+{\varepsilon }_{2}\right)}{{\varepsilon }_{1}+{\varepsilon }_{2}{\omega }^{4}/{r}^{2}}\quad {\gamma }_{i}\to 0$	${r}^{2}+2{\gamma }_{l}{\omega }^{2}$
(3)	✓	⨯	✓	$r\,\tanh (r)=\frac{{\omega }^{2}}{1+{\gamma }_{l}/{r}^{2}({\omega }^{2}-{\gamma }_{l})}$	${r}^{2}+2{\gamma }_{l}{\omega }^{2}-{\gamma }_{l}^{2}$
(4)	⨯	⨯	✓	$r\,\tanh (r)=\frac{{\omega }^{2}}{1+{\gamma }_{l}/{r}^{2}({\omega }^{2}-{\gamma }_{l})}$	${r}^{2}-{\gamma }_{l}^{2}$
(5)	✓	⨯	⨯	$r\,\tanh (r)={\omega }^{2}$	${r}^{2}+2{\gamma }_{l}{\omega }^{2}$
(6)	⨯	⨯	⨯	$r\,\tanh (r)={\omega }^{2}$	${r}^{2}$

The analysis carried out in this study confirms that the standard solution, i.e. neglecting the effects of compressibility, elasticity and gravity, may still result in satisfactory calculations of the tsunami arrival times for shallow water ($h < 2$ km) or short waves (less than 10 min period). However, for longer waves, the full solution, which considers compressibility, elasticity and gravity, becomes essential where a deeper portion of the sea bottom interacts with the ocean. For the rigid bottom case, water incompressibility is responsible for an increase in the phase speed by ${\gamma }_{l}$ while neglecting gravity decreases the phase speed by ${\gamma }_{l}/2$ of full solution in shallow water limit, $kh\ll 1$. Unlike compressible ocean over rigid bottom assumption where the phase speed approaches to an asymptote for shallow water limit, the role of bottom elasticity is proportional to the frequency of waves. Thus, at the limit of large wavelength waves the contribution of elasticity overcomes that of compressibility. The analysis depicted in Fig. 2c shows that elasticity is negligible for waves with periods smaller than 5 min, whereas it overtakes compressibility for wave periods longer than 60 minutes. On the other hand, ignoring gravity leads to an underestimate of the phase speed. Note that changes within the range of the Preliminary Reference Earth Model (PREM) parameters, taken from ref.^31,32^ for the crust and ocean, result in variations in the phase speed that are less than $0.2 \%$.

**Figure 3:**
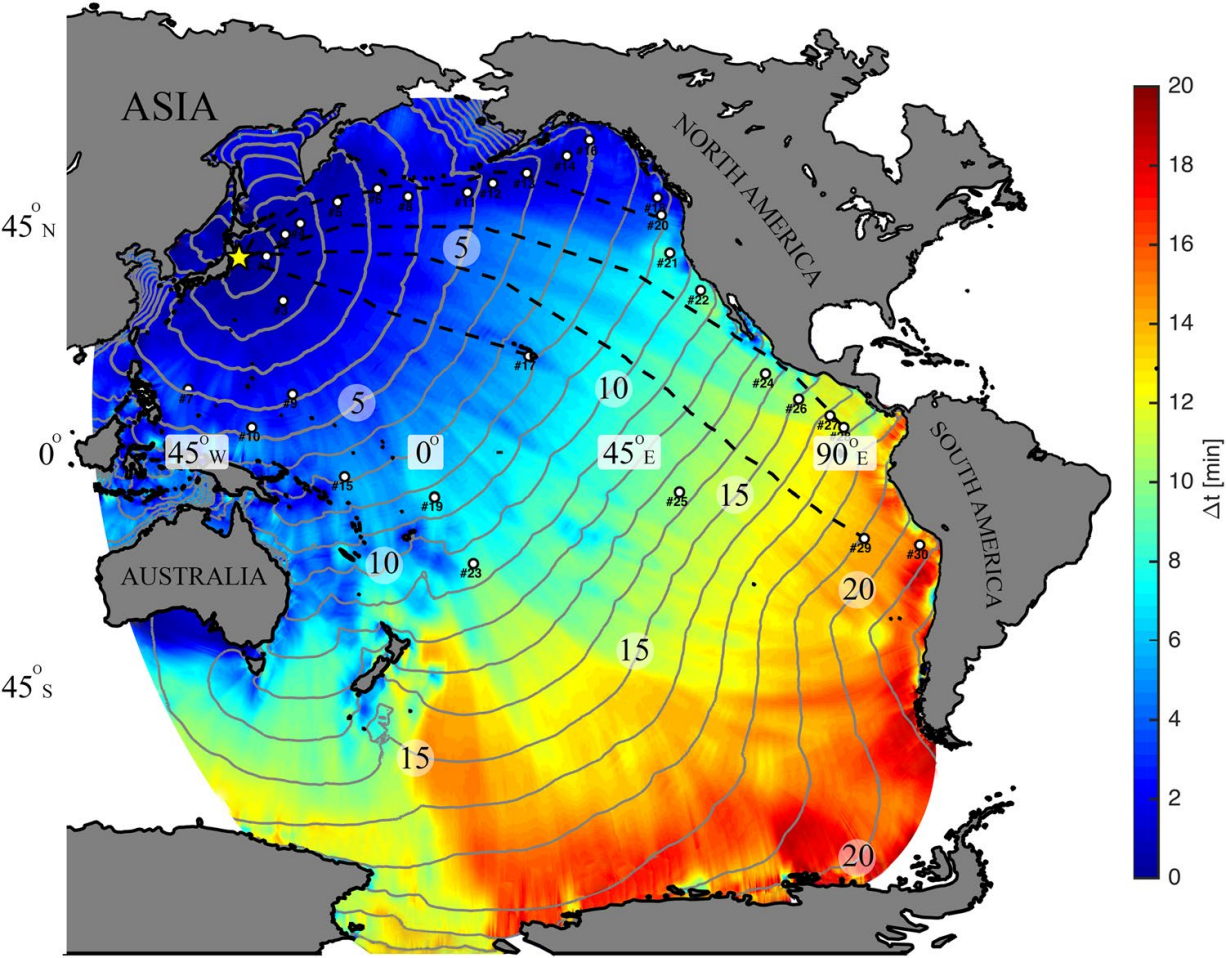
Arrival overestimation (min) between the full solution (Eq. 1) and standard dispersion relation for the case of incompressible ocean over rigid bottom. The solid contours show the hourly tsunami wave front (Period of 35 min) propagating with full solution. The source (yellow star) is Tohoku Oki 2011 event (142${}^{\circ }$22′8″E, 38${}^{\circ }$19′19″N) generated on March 11, at 14:46 local time (JST). The black dashed lines are the shortest path from Tohoku Oki Epicenter to 4 DART buoys $\#$32411 (28), $\#$32412 (29), $\#$46404 (20) and $\#$51407 (17). The DART observatories are shown with white circles.

**Figure 4:**
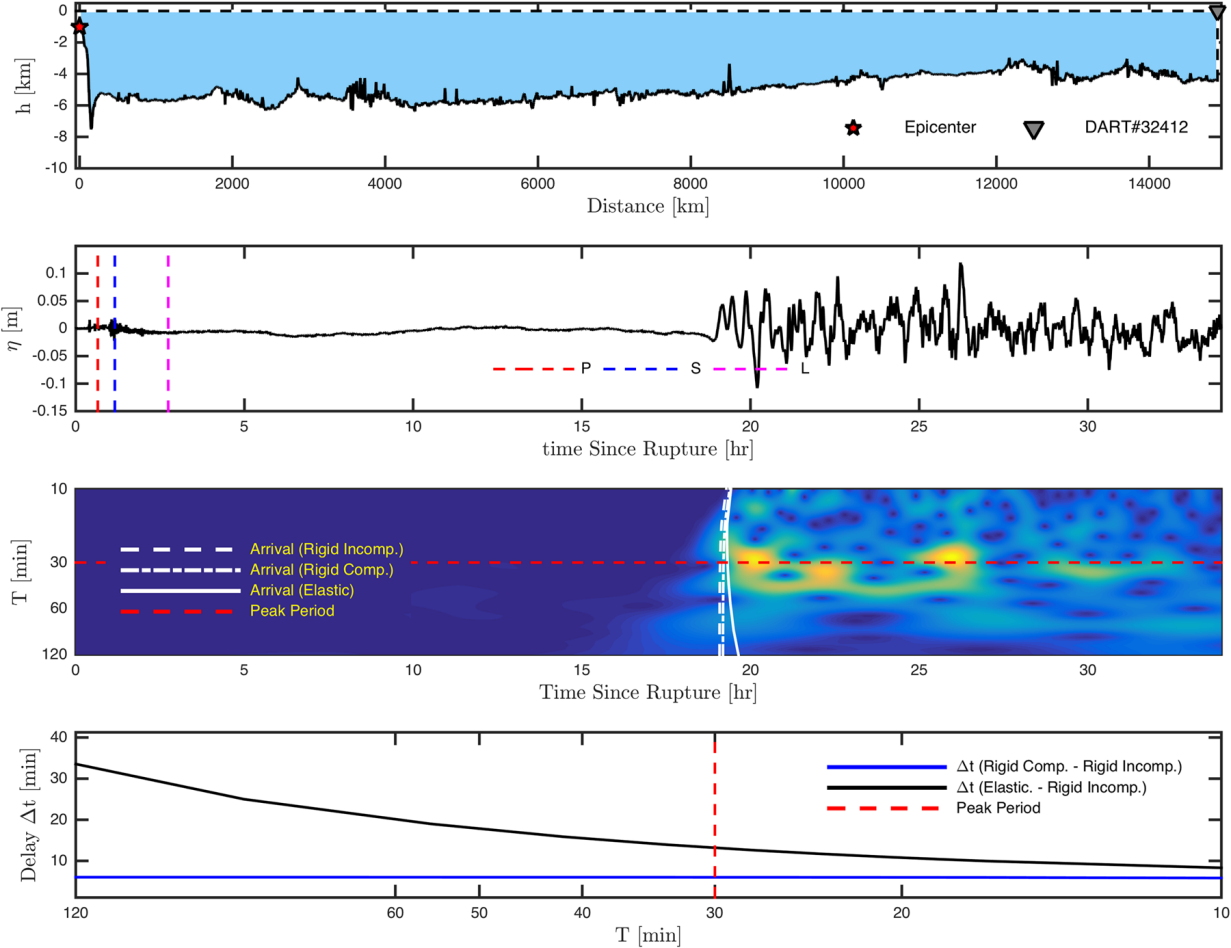
(**a**) Transect from Tohoku Oki epicenter to DART $\#$ 32411 along shortest path; (**b**) De-tided *in-situ* data (black), arrival time of compression waves in solid (P), shear waves in solid (S) and compression waves in water (L); (**c**) Spectrum density based on Wavelet analysis to determine wave periods. White lines are arrival time of signal for the full solution (solid: table 1$\#$1), neglecting elasticity and gravity (dotted: table 1$\#$5) and without compressibility, elasticity and gravity (dashed: table 1$\#$6). Red line represents peak period of leading wave; (**d**) Early arrival ($\Delta t$) due to ocean incompressibility and neglecting gravity (blue) and due to elasticity and ocean incompressibility and neglecting gravity (black). The dashed vertical red line represents peak period of leading wave from panel (c).

As a case study, we consider the Tohoku Oki 2011 tsunami^26,27,33,34^. Analyzing the frequency spectrum of the *in-situ* measurements reveals a range of wave period (10–90 min) for a 4 hr window near 1${}^{st}$ peak with a mean of $\sim$35 min at DART buoys (Fig. 1a,b). In the present study, the mean of peak periods is considered when calculating the arrival times of tsunami front based on the solution of Eq. (1) where water compressibility, bottom elasticity and gravity are all considered. In contrast and similar to the conventional tsunami models where the ocean is treated as an incompressible medium on a rigid bottom, the arrival time is calculated and compared to the full solution as shown in Fig. 3 (see supplementary file). Calculations of the phase speed reported in literature (i.e. refs^5,27^ as shown in Fig. 1d) overestimate observations, with a discrepancy reaching 30 minutes for waves with 90 min period in some regions $\sim$15000 km away from the source and close to South America (i.e. see Fig. 4d). However, considering compressibility, elasticity, and gravity results in a noticeable improvement of the arrival times at all observation locations (Figs 1c and 3). Note that the tsunami wave is a superposition of waves with different frequencies, travelling with corresponding phase speeds. As a result, the modulation would change if the correct phase speed is considered, leading to a more reliable model output.

The results of this study outline the contribution of ocean compressibility, bottom elasticity and static compression of the ocean under gravity on the propagation speed of waves, generated by tsunamigenic events ranging from 10–180 minutes periods. Parametric analysis, comparing existing ocean circulation models with the proposed full solution, reveals up to 1.3% and 4$\%$ increase in phase speed due to neglecting water compressibility and sea bottom elasticity, respectively, and up to 1$\%$ reduction in speed due to neglecting the effects of field gravitational potential. The lower the frequency of the wave, the higher the discrepancy between the full solution and standard models become, leading to earlier arrival of signals at far-field observatories, as well as changes in the signal modulation. Proper consideration of these parameters would lead to a better understanding of an interactive environment comprising a compressible ocean and an elastic earth system for a variety of waves travelling at the sea surface such as tsunami, or propagate in deep water, e.g. ocean acoustics, or in the earth crust, such as P, S, Rayleigh and Love.

There has been an increased requirement for a multi-components early warning system due to the severity of impact of such frequent events in recent decades. A reliable system should have the capability to correlate precursors and destructive tail via accurate dynamic interactions between media. The findings here not only give a better measure for the phase speed that is essential for reliable warning systems, but also construct the basic pillars of the next era of research on the propagation of waves in the ocean where water compressibility, sea-bottom elasticity, and field gravity should all be considered to better understand the physical processes involved. These should have a direct impact on major fields within geosciences, physical oceanography, and natural hazards.

### Coefficients of full dispersion relation (Dimensionless)

3 $${\varepsilon }_{1}=\frac{4\mu {k}^{2}(s+{\gamma }_{s})(q+{\gamma }_{p})}{{k}^{2}+{s}^{2}+2s{\gamma }_{s}+{\gamma }_{s}^{2}}+\lambda {k}^{2}-(\lambda +2\mu )\left({q}^{2}+2{\gamma }_{p}q+2{\gamma }_{p}^{2}\right)$$

4 $${\varepsilon }_{2}=(q+{\gamma }_{p})\frac{{k}^{2}-{s}^{2}-2s{\gamma }_{s}-{\gamma }_{s}^{2}}{{k}^{2}+{s}^{2}+2s{\gamma }_{s}+{\gamma }_{s}^{2}}$$

5 $$\beta ={\omega }^{2}({\varepsilon }_{1}-{\varepsilon }_{2})-{\gamma }_{l}{\varepsilon }_{1}$$

and

6 $${r}^{2}={k}^{2}-2{\gamma }_{l}{\omega }^{2}+{\gamma }_{l}^{2};\qquad {q}^{2}={k}^{2}-2{\gamma }_{p}{\omega }^{2}+{\gamma }_{p}^{2};\qquad {s}^{2}={k}^{2}-2{\gamma }_{s}{\omega }^{2}+{\gamma }_{s}^{2}$$

where $\lambda$ and $\mu$ are the Lame’s constants, $r$, $q$, and $s$ are the eigenvalues of the three differential equations in Eq. (1), respectively.

### Calculations of tsunami travel time

The tsunami travel time is calculated from the epicentre for a given frequency using dispersion relations presented in Table 1 ($\#1$ and $\#6$). Via a time marching scheme, the furthest points the front wave can reach within $\Delta t$ is calculated considering depth dependent phase speed and distance from front wave. The computation starting point is the epicentre, at ${t}_{0}=0$, corresponding to rupture time and calculating the coverage of tsunami for the succeeding time steps until either the tsunami covers the whole domain or reaches the coast. The Haversine method is used to convert spacing between WGS84 degrees and planar meters. For Tohoku event, we have considered ${t}_{0}=0$ on March 11, 2011 at 14:46 (JST) and $\Delta t=60$ s for 24 hrs. Employing a tsunami travel time estimator, the front wave of a long wavelength gravity wave with the same average period, $\sim 35$ min, of the Tohoku 2011 tsunami (see Fig. 1) is shown in Fig. 3.

## Supplementary information

Supplementary Information

## Data Availability

Correspondence and requests for materials should be addressed to Ali Abdolali. (email: ali.abdolali@noaa.gov).
