# Supplementary material for: Effect of Water Compressibility, Sea-floor Elasticity, and Field Gravitational Potential on Tsunami Phase Speed
Source: Sci Rep. 2019 Nov 14;9:16874. doi: 10.1038/s41598-019-52475-0 (PMC6856086; doi:10.1038/s41598-019-52475-0)
Supplement: Supplementary file 1 — Supplementary Information [file 41598_2019_52475_MOESM1_ESM.pdf]

Supplementary materials for:

Effect of Water Compressibility, Sea-floor Elasticity, and Field Gravitational Potential on Tsunami Phase Speed

Ali Abdolali[1,2], Usama Kadri[3.4] & James T. Kirby[5]

Governing equations and boundary conditions

The analysis made in this supplementary report is dimensional. In the main manuscript all quantities in where normalized using the water depth h is a length scale, $\sqrt{h/g}$ as a timescale, and densities were normalized by the water density ρ_l, which was made unity. The governing equations for inviscid motion of a compressible medium, i, in a three dimensional Cartesian coordinate system are given by

$$\begin{aligned} &\rho_{i,t} + \nabla \cdot (\rho_i \mathbf{u}) = 0 \\ &\mathbf{u}_t + (\mathbf{u} \cdot \nabla)\mathbf{u} + \frac{1}{\rho_i}\nabla p = -g\mathbf{i}_z \end{aligned} \tag{1}$$

where g is the gravitational acceleration, ρ_i is the medium density, p is the pressure and $\mathbf{u}$ is the velocity. The problem is formulated in terms of velocity potential ($\mathbf{u} = \nabla\phi_i$), provided that there is a relation between pressure and density, and assuming irrotationality of the medium. The relation between pressure and density variations can be expressed by $d\rho_i = (d\rho_i/dp)dp = dp/c_i^2$, where c_i

[1]National Oceanic and Atmospheric Administration (NOAA), College Park MD 20740 USA
[2]University Corporation for Atmospheric Research (UCAR), Boulder, CO 80301 USA
[3]School of Mathematics, Cardiff University, Cardiff, CF24 4AG, UK
[4]Department of Mathematics, Massachusetts Institute of Technology, Cambridge, MA 02139, USA
[5]Department of Civil and Environmental Engineering, University of Delaware, Newark, DE 19716, USA

is speed of sound in the medium which is taken constant.

The undisturbed density ($\rho_{i,0}$) and pressure ($p_{i,0}$) can be defined as

$$\rho_{i,0}(z) = \rho_{i,s} \mathrm{e}^{-2\gamma_i z}; \quad p_{i,0}(z) = \frac{\rho_{i,s} g}{2\gamma_i} \left(\mathrm{e}^{-2\gamma_i z} - 1 \right); \quad \mathbf{u} = 0 \tag{2}$$

where $\rho_{i,s}$ and $p_{i,s}$ are density and pressure at medium surface respectively and and $\gamma_i = g/2c_i^2$ is the lapse rate for density and pressure with elevation. After linearizing about the basic state, the equation governing ϕ_i is given by Ref.[1]

$$\phi_{i,tt} - c_i^2 (\nabla_h^2 \phi_i + \phi_{i,zz}) = -g \phi_{i,z} \tag{3}$$

Equation (3) follows from the use of the complete expressions (2) for background density and pressure; however, in practice these are well approximated by the leading order expressions retaining compressibility effects:

$$\rho_{i,0}(z) = \rho_{i,s}(1 - 2\gamma_i z); \quad p_{i,0}(z) = -\rho_{i,s} g z \,(1 - \gamma_i z) \tag{4}$$

In this study, water is treated as an inviscid barotropic fluid with constant sound speed, c_l, and the motion is assumed irrotational. On the other hand, the solid layer is treated as an elastic half space that undergoes rotation and compression with constant pressure and shear wave speeds, c_p and c_s. The problem can be expressed by three-wave equation in the form of a velocity potential, ϕ_l in water (inviscid and irrotational), a dilatation irrotational potential, ϕ_s and a rotation potential

with stream function, ψ_s in the solid sea-floor,

$$\left.\begin{aligned}
L(\phi_l) &= \frac{\partial^2 \phi_l}{\partial t^2} - c_l^2(\nabla_h^2 \phi_l + \phi_{l,zz}) + g\phi_{l,z} = 0 \qquad -h + \eta_2(x,y,t) \le z \le \eta_1(x,y,t) \\
L_p(\phi_s) &= \frac{\partial^2 \phi_s}{\partial t^2} - c_p^2(\nabla_h^2 \phi_s + \phi_{s,zz}) + g\phi_{s,z} = 0 \qquad z \le -h + \eta_2(x,y,t) \\
L_s(\psi_s) &= \frac{\partial^2 \psi_s}{\partial t^2} - c_s^2(\nabla_h^2 \psi_s + \psi_{s,zz}) + g\psi_{s,z} = 0 \qquad z \le -h + \eta_2(x,y,t)
\end{aligned}\right\} \tag{5}$$

∇_h^2 is the horizontal Laplacian and subscripts on dependent variables denote partial derivatives. The interfacial displacements η_1 and η_2 represent response of the free surface and layer interface to hydro-acoustic disturbances. c_p and c_s are related to the Lame's elasticity constants (λ, μ) and the earth density (ρ_s).

$$c_p = \sqrt{(\lambda + 2\mu)/\rho_s} \tag{6}$$

$$c_s = \sqrt{\mu/\rho_s} \tag{7}$$

The horizontal and vertical velocities in fluid are

$$\dot{U} = -\frac{\partial \phi_l}{\partial x}; \quad \dot{W} = -\frac{\partial \phi_l}{\partial z} \tag{8}$$

The horizontal and vertical velocities in the solid earth is

$$\dot{U}_s = \frac{\partial \phi_s}{\partial x} + \frac{\partial \psi_s}{\partial z}; \quad \dot{W}_s = \frac{\partial \phi_s}{\partial z} - \frac{\partial \psi_s}{\partial x} \tag{9}$$

and the linearized boundary condition at the surface is

$$\frac{\partial^2 \phi_l}{\partial t^2} + g\frac{\partial \phi_l}{\partial z} = 0 \qquad z = 0 \tag{10}$$

There are three boundary conditions at the solid earth interface ($z = -h$):

$$\left.\begin{aligned} &\dot{W}_s = -\frac{\partial \phi_l}{\partial z} \qquad \text{Kinematic} \\ &\sigma_{zz} = -P_{l,t} \qquad \text{Dynamic} \\ &\sigma_{xz} = 0 \qquad \text{Zero tangential stress} \end{aligned}\right\} \tag{11}$$

From Hooke's law of elasticity we can write

$$\sigma_{zz} = \lambda(\frac{\partial \dot{U}_s}{\partial x} + \frac{\partial \dot{W}_s}{\partial z}) + 2\mu \frac{\partial \dot{W}_s}{\partial z} \tag{12}$$

$$\sigma_{xz} = \mu(\frac{\partial \dot{W}_s}{\partial x} + \frac{\partial \dot{U}_s}{\partial z}) \tag{13}$$

and dynamic pressure in fluid

$$P_l = \rho_l \frac{\partial \phi_l}{\partial t} \tag{14}$$

where ρ_l is fluid density. For the case of a flat bottom, the velocity potentials for a given angular frequency ω may be expanded in terms of plane waves

$$\left.\begin{aligned} &\phi_l(x, y, z, t) = \sum_{n=0}^{\infty} \tilde{M}_n(z) e^{i(kx - \omega t)} \qquad -h \le z \le 0 \\ &\phi_s(x, y, z, t) = \sum_{n=0}^{\infty} \tilde{N}_n(z) e^{i(kx - \omega t)} \qquad z \le -h \\ &\psi_s(x, y, z, t) = \sum_{n=0}^{\infty} \tilde{P}_n(z) e^{i(kx - \omega t)} \qquad z \le -h \end{aligned}\right\} \tag{15}$$

where k_n are the modal wave numbers. Substituting (15) in (5) and (10)-(11) with h constant gives the boundary value problem (BVP)

$$\left.\begin{aligned} &\tilde{M}'' - 2\gamma_l \tilde{M}' - \tilde{r}^2 \tilde{M} = 0 \qquad -h \le z \le 0 \\ &\tilde{N}'' - 2\gamma_p \tilde{N}' - \tilde{q}^2 \tilde{N} = 0 \qquad z \le -h \\ &\tilde{P}'' - 2\gamma_s \tilde{P}' - \tilde{s}^2 \tilde{P} = 0 \qquad z \le -h \end{aligned}\right\} \tag{16}$$

where $\gamma_l = g/2c_l^2$, $\gamma_p = g/2c_p^2$, $\gamma_s = g/2c_s^2$ and the separation constants for the case of density/pressure fluctuations in an otherwise constant density ocean and elastic earth are

$$\left.\begin{aligned} \tilde{r}^2 &= k^2 - \frac{\omega^2}{c_l^2} \\ \tilde{q}^2 &= k^2 - \frac{\omega^2}{c_p^2} \\ \tilde{s}^2 &= k^2 - \frac{\omega^2}{c_s^2} \end{aligned}\right\} \tag{17}$$

The problem is put in Sturm-Liouville form by the substitution:

$$\left.\begin{aligned} \tilde{M}(z) &= Me^{\gamma_l z} \\ \tilde{N}(z) &= Ne^{\gamma_p z} \\ \tilde{P}(z) &= Pe^{\gamma_s z} \end{aligned}\right\} \tag{18}$$

Therefore (16) becomes

$$\left.\begin{aligned} M'' - r^2 M = 0 \\ N'' - q^2 N = 0 \\ P'' - s^2 P = 0 \end{aligned}\right\} \tag{19}$$

with

$$\left.\begin{aligned} r^2 &= \tilde{r}^2 + {\gamma_l}^2 \quad \Rightarrow r^2 = k^2 - \tfrac{\omega^2}{c_l^2} + {\gamma_l}^2 \\ q^2 &= \tilde{q}^2 + {\gamma_p}^2 \quad \Rightarrow q^2 = k^2 - \tfrac{\omega^2}{c_p^2} + {\gamma_p}^2 \\ s^2 &= \tilde{s}^2 + {\gamma_s}^2 \quad \Rightarrow s^2 = k^2 - \tfrac{\omega^2}{c_s^2} + {\gamma_s}^2 \end{aligned}\right\} \tag{20}$$

Following Ref.[2] for eigenfunctions, normalized to a value of unity at $z = 0$

$$M(z) = \cosh(rz) + \mathcal{X}\sinh(rz) = \cosh(rz) + \left(\frac{\omega^2}{rg} - \frac{\gamma_l}{r}\right)\sinh(rz) \tag{21}$$

$$N(z) = -e^{(q(h+z))}\frac{r}{q + 2\gamma_p} \tag{22}$$

$$\frac{k^2+s^2+2s\gamma_s+\gamma_s^2}{k^2-s^2-2s\gamma_s-\gamma_s^2}[(\sinh(rh)-\mathcal{X}\cosh(rh))+\frac{\gamma}{r}(\mathcal{X}\sinh(rh)-\cosh(rh))] \tag{23}$$

$$P(z)=e^{(s(h+z))}\frac{2irk}{k^2-s^2-2s\gamma_s-\gamma_s^2}(\sinh(rh)-\mathcal{X}\cosh(rh))+\frac{\gamma}{r}(\mathcal{X}\sinh(rh)-\cosh(rh)) \tag{24}$$

where $\mathcal{X}=\omega^2/rg-\gamma_l/r$. The dispersion relation governing r is given by (25)

$$\tanh(rh)=\frac{\frac{\omega^2}{r}\left([q+\gamma_p]\rho_l\left(\frac{k^2-s^2-2s\gamma_s-\gamma_s^2}{k^2+s^2+2s\gamma_s+\gamma_s^2}\right)+\frac{1}{g}\left(\frac{4\mu k^2[s+\gamma_s][q+\gamma_p]}{k^2+s^2+2s\gamma_s+\gamma_s^2}+(\rho_s\omega^2-2\mu k^2)-2\gamma_p(q+\gamma_p)(\lambda+2\mu)\right)\right)}{\frac{\mathcal{X}\omega^2\rho_l[q+\gamma_p]}{r}\left(\frac{k^2-s^2-2s\gamma_s-\gamma_s^2}{k^2+s^2+2s\gamma_s+\gamma_s^2}\right)+[1+\frac{\gamma_l}{r}\mathcal{X}]\left(\frac{4\mu k^2[s+\gamma_s][q+\gamma_p]}{k^2+s^2+2s\gamma_s+\gamma_s^2}+(\rho_s\omega^2-2\mu k^2)-2\gamma_p(q+\gamma_p)(\lambda+2\mu)\right)} \tag{25}$$

From here, it is easy to see that the non dimensional form of the general dispersion relation is relation (2) with coefficients (3)-(5) of the main manuscript.

For $\gamma_l=\gamma_p=\gamma_s=0$ and $\tilde{r}=r$, $\tilde{q}=q$ and $\tilde{s}=s$, dispersion relation reduce to the form of compressible ocean with an elastic half space as defined in Ref.[2]

$$\tanh(rh)=\frac{\frac{\omega^2}{r}\left(q\rho_l\left(\frac{k^2-s^2}{k^2+s^2}\right)+\frac{1}{g}\left(\frac{4k^2qs\mu}{k^2+s^2}-((\lambda+2\mu)q^2-\lambda k^2)\right)\right)}{\frac{\omega^4 q\rho_l}{gr^2}\left(\frac{k^2-s^2}{k^2+s^2}\right)+\left(\frac{4k^2qs\mu}{k^2+s^2}-((\lambda+2\mu)q^2-\lambda k^2)\right)} \tag{26}$$

The phase speed of surface gravity waves can be evaluated from the dispersion relations (25 & 26), where $k\geq\omega/c_l\geq\omega/c_s\geq\omega/c_p$ for progressive surface tsunami waves ($k^2>0$) and evanescent compression waves in water and compression wave and shear wave in solid earth (r, q and s are real). Note that the solution is limited to values of $k^2>0$ where there is no interaction with trapped modes in a spatially uniform domain.

If elasticity is ignored, retaining water compressibility and gravity terms, the dispersion relation Eq. (3.2) of Ref.[3] or Eq. (11) of Ref.[4] are retrieved. Neglecting elasticity and gravity leads to the standard dispersion relation $\omega^2=gr\tanh(rh)$.

Reference

1. Bondi, H. Waves on the surface of a compressible liquid. *Mathematical Proceedings of the Cambridge Philosophical Society* **43**, 7595 (1947).

2. Eyov, E., Klar, A., Kadri, U. & Stiassnie, M. Progressive waves in a compressible-ocean with an elastic bottom. *Wave Motion* **50**, 929 – 939 (2013).

3. Kadri, U. & Stiassnie, M. Generation of an acoustic-gravity wave by two gravity waves, and their subsequent mutual interaction. *Journal of Fluid Mechanics* **735**, R6 (2013).

4. Abdolali, A. & Kirby, J. T. Role of compressibility on tsunami propagation. *Journal of Geophysical Research: Oceans* **122**, 9780–9794 (2017).
